# The clinical significance of plasma sCD25 as valuable biomarker for progression and prognosis of tuberculosis

**DOI:** 10.1186/s12879-023-08798-5

**Published:** 2024-01-22

**Authors:** Xin Yu, Yayan Niu, Junchi Xu, Xiaolong Zhang, Haiyan Wu, Yuhan Wang, Jianping Zhang, Meiying Wu

**Affiliations:** 1 Department of Tuberculosis, The Fifth People’s Hospital of Suzhou, The Affiliated Infectious Diseases Hospital of Soochow University, Suzhou, China; 2 Department of Clinical laboratory, The Fifth People’s Hospital of Suzhou, Suzhou, China; 3 Department of Tuberculosis, Suzhou Center for Disease Control and Prevention, Suzhou, China

**Keywords:** Tuberculosis, Plasma sCD25, Progression, Prognosis, Treatment outcome

## Abstract

**Background:**

sCD25 is an important immune molecule for T cell regulation. Tracking the detection of plasma sCD25 plays an important role in the evaluation of immune function, progression, and prognosis of tuberculosis (TB) patients. This study analyzed the association of plasma sCD25 levels with clinical, laboratory, CT imaging characteristics, and clinical outcome of TB patients.

**Methods:**

The clinical data of 303 TB patients treated in the Fifth People’s Hospital of Suzhou from October 2019 to January 2022 were retrospectively analyzed. The levels of sCD25 in plasma were detected by ELISA. According to the cut-off threshold of plasma sCD25 levels, the patients were divided into a low-value group (Group TB1) and a high-value group (Group TB2). The association of plasma sCD25 levels with clinical, laboratory, and CT imaging characteristics of TB patients, as well as their TB treatment outcome were analyzed.

**Results:**

The levels of plasma sCD25 of patients with TB patients were higher than that of the healthy control group (*P* < 0.01). Among the 303 TB patients, the levels were increased in Group TB2 patients (0.602 ± 0.216 vs. 1.717 ± 0.604 ng/ml, *P* < 0.001), and there was a progressive reduction after anti-TB treatment. Furthermore, patients in Group TB2 showed higher positive rates in sputum smear (52.0% vs. 34.3%; *P* = 0.003), sputum culture (69.7% vs. 56.9%; *P* = 0.032), Xpert MTB/RIF (66.3% vs. 51.2%; *P* = 0.013) and TB-DNA (51.5% vs. 31.2%; *P* = 0.001) than those in Group TB1. Patients in Group TB2 had higher incidence in cough (78.8% vs. 62.3%; *P* = 0.004), expectoration (64.4% vs. 45.1%; *P* = 0.001), concomitant extrapulmonary TB (14.1% vs. 5.9%; *P* = 0.016), cavities (47.9% vs. 34.0%; *P* = 0.022), and unfavorable outcomes after anti-TB treatment.

**Conclusion:**

The clinical, laboratory and radiological manifestations of TB patients with high plasma sCD25 levels indicate that the disease is more severe. Tracking plasma sCD25 detection of TB patients has evident clinical significance. It is noteworthy that when the plasma sCD25 levels are significantly elevated, patients should be cautious of the TB progression and disease severity.

**Supplementary Information:**

The online version contains supplementary material available at 10.1186/s12879-023-08798-5.

## Introduction

Tuberculosis (TB), a communicable disease caused by the bacillus *Mycobacterium tuberculosis* (MTB), is a major cause of ill health and one of the leading causes of death worldwide each year [1]. According to the 2022 Global Tuberculosis Report, there are approximately 10.6 million new TB patients worldwide and 1.6 million deaths [2]. China is one of the Top 30 high TB burden countries, ranking the third place in the number of TB cases. The estimated number of new TB patients in 2021 is 780,000 and the incidence rate is 55/100,000 [2]. However, there is still a large gap between the estimated number of new TB cases globally and the newly diagnosed cases, with 4.2 million TB patients in 2021 either undiagnosed or not formally reported to the national authorities. Generally, the onset of TB clinical symptoms is insidious, and the disease progression is slow. The duration of symptoms prior to diagnosis may range from 2 weeks to several years [3]. Therefore, delays in diagnosis of TB are common, which is the biggest obstacle to TB control. Additionally, the clinical indicators of tuberculosis cure, including negative sputum culture and absorption of chest lesions, are often lagging. In this regard, it may be clinically attractive and meaningful to search for a valuable biomarker that can be used for monitoring the progress and treatment outcome of TB.

After MTB infection, cell-mediated immunity plays an important role in the occurrence, progression, and prognosis of TB. Interleukin 2 (IL-2) can directly regulate the bactericidal activities of immune cells, and regulate the proliferation of effector T cells, further activating the bactericidal function in the anti-MTB immune response [4–6]. Signal transduction of IL-2 is mediated via IL-2 receptor (IL-2R). IL-2R complex has 3 distinct subunits, namely designated IL-2Rα, IL-2Rβ, and IL-2Rγ, respectively [7]. IL-2Rα, also known as CD25, is expressed by Treg, but is also induced upon T-cell activation and generally considered as a marker of T cell activation [8]. The expression of CD25 on T cells and its interaction with IL-2 are pivotal features of immune responsiveness.

The T cells expression of CD25 is significantly increased in TB patients. Although flow cytometry CD25 analysis cannot distinguish active from latent infection, it has been used to identify clinically MTB infected [9–11]. sCD25 results from the proteolytic cleavage of the ectodomains in the membrane-bound IL-2Rα chain [8]. sCD25 is abundantly found in the circulation of healthy individuals and further increased in a variety of diseases including autoimmune diseases, cancers, and inflammatory diseases, which has led to several studies analyzing its potential as a diagnostic marker [12–14]. The level of sCD25 increases in patients with extensive granulomatous lesions [15–18]. In fact, in sarcoidosis and TB, elevated levels of sCD25 have been reported and are effective markers for measuring disease activities [18–20].

The expression levels of sCD25 are elevated in tuberculosis, but the association of plasma sCD25 levels with the disease severity, progression and prognosis of TB patients are not completely clear. In this study, we have examined the plasma sCD25 levels in patients with pulmonary TB and the changes following anti-TB chemotherapy. Then we analyzed the association of plasma sCD25 levels with clinical, laboratory, CT imaging characteristics, and the clinical outcome of TB patients. This study provides immunological indicators for the evaluation of the immune status of TB patients, and even biomarkers for the disease severity, progression, and prognosis of TB.

## Methods

### Subjects and inclusion criteria

Pulmonary TB patients were recruited from the Tuberculosis Department at the Fifth People’s Hospital of Suzhou between October 2019 and January 2022. A total of 57 healthy controls (HC), sex and age-matched to the patients, were studied concurrently (31 males and 26 females; median age was 33 years with interquartile range of 18–67 years). The research related to human use complied with all the relevant national regulations and institutional policies in accordance with the tenets of the Helsinki Declaration and was approved by the Ethics Committee of the Fifth People’s Hospital of Suzhou.

The diagnosis of pulmonary TB was confirmed by positive MTB microbiologic results, or clinical and CT imaging. Subjects with lesions in over three lung fields, with respiratory symptoms such as cough, expectoration and chest tightness, and a body temperature ≥ 39 °C were considered as severe TB patients, as described previously [21]. In contrast, those with mild illness were in the mild TB group. Patients with other infectious and pulmonary diseases, such as asthma and lung cancer, and those who take medicine glucocorticoids and other immunosuppressants were excluded from the study.

### CT image and laboratory examination

Pulmonary TB were examined with spiral CT, and the imaging results were analyzed by two physicians who specialized in CT examination, and the CT imaging characteristics were recorded. Sputum samples were routinely tested to diagnose pulmonary TB, including microscopy examination, mycobacterial culture, and Xpert MTB/RIF. In addition, QuantiFERON (QFT) IGRA (Qiagen, Venlo, Limburg, Netherlands) was offered as an immunological test for the diagnosis of TB.

### Measurement of plasma sCD25

Whole blood was taken from the patients before anti-TB treatment as well as after 3, 6, and 12 months of the treatment. Blood from healthy subjects was collected as control. Plasma was separated from the whole blood after centrifugation at 2000×g for 15 min and stored at − 80 °C before sCD25 assay. Plasma levels of sCD25 (Xvguang Kexing Antibody Biotechnology Co., Ltd) were examined by enzyme-linked immunosorbent assay (ELISA) according to the manufacturers’ instructions. All samples were tested in the same batch to minimize intraassay variability.

According to the cut-off threshold of plasma sCD25 levels, the TB patients were divided into a low-value group and a high-value group. Those whose plasma sCD25 values were below the cut-off threshold were in the low-value group (Group TB1), and those above were in the high-value group (Group TB2).

### Statistical analysis

The statistical analysis was done using GraphPad Prism 9.0 (GraphPad Inc., La Jolla, USA) and SPSS 26.0 (SPSS Inc., Chicago, IL, United States). Data are presented in mean ± standard deviation (SD) or Median (interquartile range, IQR). The Mann–Whitney U test and Student’s t test were used for continuous variables, and the Pearson chi-square test was used to compare categorical variables. *P* values < 0.05 were considered statistically significant.

The receiver operator characteristic (ROC) curves analysis was performed using 303 TB patients and 57 healthy controls. The binary classification criteria we used was whether the patient had TB (where 1 represented TB patients and 0 represented healthy controls). After determining the optimal cutoff value for sCD25, we classified 303 TB patients based on their sCD25 levels. Among them, 204 patients with sCD25 levels below this cutoff value assigned 0, and 99 patients with sCD25 levels above or equal to this cutoff value assigned 1 were evaluated by Logistic regression model analysis to evaluate which clinical variables were significantly associated with higher sCD25 levels.

## Results

### Patients

**Fig. 1:**
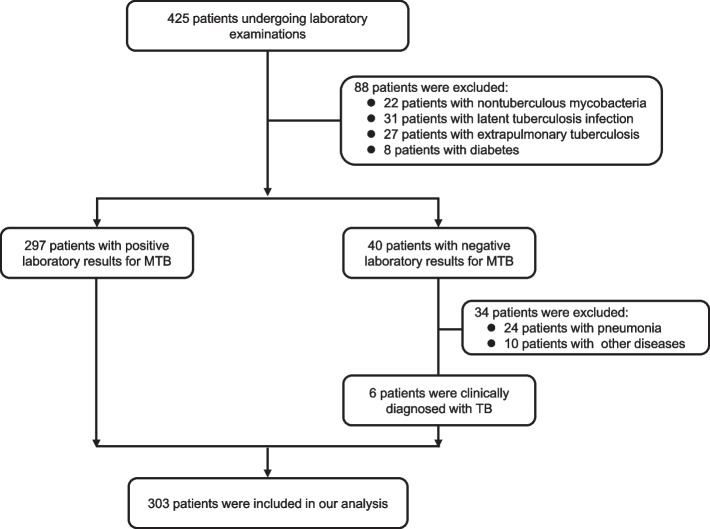
Flow diagram of the selection of patients for inclusion

A total of 425 patients with indicators suggestive of pulmonary TB were screened in our study, and 122 (28.7%) were excluded, leaving 303 TB cases included in the final analysis. Among the 303 patients, 297 (98.0%) were positive in MTB laboratory tests, and the other 6 (2.0%) were clinically diagnosed with TB case (Fig. 1).

Among the 303 patients we studied, there were 174 (57.4%) males and 129 (42.6%) females, and the median age at onset was 32 years (18–68 years). The most frequent clinical signs were cough in 205 (67.6%) patients, expectoration in 156 (51.4%), night sweat in 93 (30.6%), fatigue in 55 (18.1%), and 52 (17.1%) had fever signs. 65% of patients had comorbidity, of which bronchial TB accounted for the highest proportion (Table 1).

**Table 1:** Demographic and clinical characteristics of patients

	Total (*n* = 303)
Sex, *n* (%)	
Male	174 (57.4)
Female	129 (42.6)
Age (years), median (range)	32 (18–68)
Age group (years), *n* (%)	
18–29	120 (39.6)
30–39	99 (32.7)
40–49	27 (8.9)
50–59	37 (12.2)
60–69	20 (6.6)
Clinical characteristics, *n* (%)	
Cough	205 (67.6)
Expectoration	156 (51.4)
Night sweat	93 (30.6)
Fatigue	55 (18.1)
Fever	52 (17.1)
Dyspnea	46 (15.1)
Chest pain	40 (13.2)
Anorexia	34 (11.2)
Hemoptysis	30 (9.9)
Weight loss	26 (8.5)
Comorbidity, *n* (%)	
Bronchial TB	122 (40.2)
Extrapulmonary TB	26 (8.5)
TB pleurisy	26 (8.5)
Hypertension	24 (7.9)

Data are given at median (full range) or number (percentage)

### Increased plasma concentrations of sCD25 in patients with TB

**Fig. 2:**
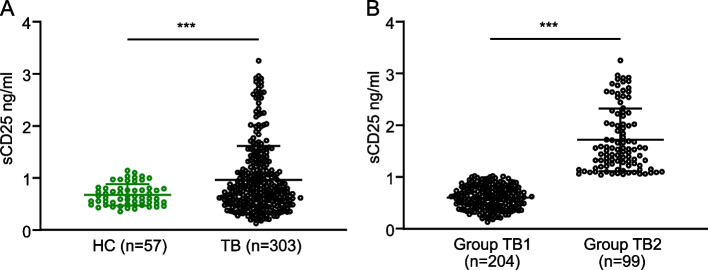
Plasma levels of sCD25 in HC and TB patients. The levels of sCD25 in plasma were detected by ELISA. **A** Plasma levels of sCD25 in healthy controls (HC) and TB patients. **B** The levels of sCD25 in the below (Group TB1) and above cut-off value group (Group TB2). Data are mean ± SD, *** *P* < 0.001

Plasma concentrations of sCD25 in healthy controls (HC) and pulmonary TB patients were tested by ELISA. The sCD25 levels in TB patients before anti-TB treatment (0.966 ± 0.651 ng/ml) were markedly raised compared to HC subjects (0.677 ± 0.204 ng/ml) (Fig. 2A). These results indicate that plasma sCD25 levels are increased in patients with TB.

We performed subgroup analysis of TB patients based on cut-off values. To select the cut-off value, the ROC curves analysis was done in this study (Additional file 1: Fig. S1, Table S1). When the plasma sCD25 concentration was 1.037 ng/ml, the maximum Youden index can be reached in the ROC curve. We divided TB patients into two groups. Those whose plasma sCD25 values were below the 1.037 ng/ml were in the low-value group (Group TB1), and those above were in the high-value group (Group TB2). Compared with Group TB1, the levels of sCD25 were significantly higher in Group TB2 (0.602 ± 0.216 vs. 1.717 ± 0.604 ng/ml, *P* < 0.001) (Fig. 2B).

### Plasma sCD25 was associated with clinical, laboratory, and CT imaging characteristics of TB patients, as well as with their TB treatment outcome

To investigate the potential clinical association of sCD25 with the TB disease, we analyzed the clinical, laboratory, and chest CT imaging characteristics of the patients in Group TB1 and Group TB2 (Table 2). Among the 303 patients, 204 were in Group TB1, with 116 (56.9%) males, and 99 were in Group TB2, with 58 males (58.6%). No significant difference in sex was observed between the two groups. Compared with Group TB1, Group TB2 had a higher percentage of relapse and severe illness, although the difference was not statistically significant. Patients in Group TB2 showed higher positive rates in sputum smear (52.0% vs. 34.3%; *P* = 0.003), sputum culture (69.7% vs. 56.9%; *P* = 0.032), Xpert MTB/RIF (66.3% vs. 51.2%; *P* = 0.013) and TB-DNA (51.5% vs. 31.2%; *P* = 0.001) than those in Group TB1. In addition, Group TB2 had higher incidence than Group TB1, in cough (78.8% vs. 62.3%; *P* = 0.004), expectoration (64.4% vs. 45.1%; *P* = 0.001), concomitant extrapulmonary TB (14.1% vs. 5.9%; *P* = 0.016), and cavities (47.9% vs. 34.0%; *P* = 0.022). However, the rate of sputum culture negative conversion of Group TB2 was significantly lower than that of Group TB1 (89.2% vs. 97.5%; *P* = 0.003). We further analyzed the results of clinical outcome of the two groups and found that the proportion of unfavorable treatment outcomes after anti-TB treatment 3 and 6 months of Group TB2 was higher than that of Group TB1.

**Table 2:** Comparison of clinical, laboratory, CT imaging characteristics and treatment outcome between Group TB1 and Group TB2

Factor	Group TB1		Group TB2		χ^2^	*P*
	n	%	n	%		
Sex					0.081	0.776
Male	116	56.9	58	58.6		
Female	88	43.1	41	41.4		
Type of case					2.768	0.96
New cases	198	97.1	92	92.9		
Relapse cases	6	2.9	7	7.1		
State of illness					33.54	0
Mild	183	89.7	61	61.6		
Severe	21	10.3	38	38.4		
Sputum smear					8.663	0.003
Negative	134	65.7	47	48.0		
Positive	70	34.3	51	52.0		
2 months smear					5.62	0.018
Negative	188	96.9	83	90.2		
Positive	6	3.1	9	9.8		
Sputum culture					4.617	0.032
Negative	88	43.1	30	30.3		
Positive	116	56.9	69	69.7		
2 months culture					4.943	0.026
Negative	188	98.4	86	93.5		
Positive	3	1.6	6	6.5		
IGRAS					2.829	0.093
Negative	13	6.6	12	12.4		
Positive	185	93.4	85	87.6		
Xpert					6.116	0.013
Negative	99	48.8	33	33.7		
Positive	104	51.2	65	66.3		
TB-DNA					11.667	0.001
Negative	139	68.8	48	48.5		
Positive	63	31.2	51	51.5		
Negative conversion rate					8.692	0.003
No	5	2.5	10	10.8		
Yes	192	97.5	83	89.2		
Cough					8.326	0.004
No	77	37.7	21	21.2		
Yes	127	62.3	78	78.8		
Expectoration					10.197	0.001
No	112	54.9	35	35.4		
Yes	92	45.1	64	64.4		
Haemoptysis					0.813	0.367
No	186	91.2	87	87.9		
Yes	18	8.8	12	12.1		
Concomitant bronchial TB					2.351	0.125
No	128	62.7	53	53.5		
Yes	76	37.3	46	46.5		
Concomitant extrapulmonary TB					5.796	0.016
No	192	94.1	85	85.9		
Yes	12	5.9	14	14.1		
Concomitant TB pleurisy					29.907	0
No	199	97.5	78	78.8		
Yes	5	2.5	21	21.2		
Lung fields					18.435	0
	141	69.1	43	43.4		
≥3	63	30.9	56	56.6		
Cavity					5.235	0.022
No	134	66.0	49	52.1		
Yes	69	34.0	45	47.9		
3 months treatment outcome					5.417	0.02
Favorable	178	93.7	81	85.3		
Unfavorable	12	6.3	14	14.7		
6 months treatment outcome					4.739	0.029
Favorable	148	98.0	69	92.0		
Unfavorable	3	2.0	6	8.0		

*P* < 0.05 is statistically significant. Abbreviations: TB, tuberculosis

After age and multiple factor adjustment, there was a significantly positive associated between Group TB2 with severe illness, positive MTB test results, lung lesions, and unfavorable treatment outcomes (Table 3). Based on multiple logistic regression analysis, compared with parameters in Group TB1, TB2 was associated with severe illness [odds ratio (OR) = 5.392, 95% CI = 2.934–9.910, *P* < 0.001]. After adjustment for other possible factors, this risk was reduced [odds ratio (OR) = 3.829, 95% CI = 1.662–9.071, *P* < 0.002], but was still significantly higher than Group TB1, and was statistically significant. These results suggest that the clinical, laboratory and radiological manifestations of TB patients with high plasma sCD25 levels indicate that the disease is more severe.

**Table 3:** Multivariate analysis of independent factors for the plasma sCD25 levels association with clinical parameters

Factor	Age adjust OR (95%CI)	*P*	Multi-factor adjust # OR(95%CI)	*P*
Severe illness	5.392 (2.934,9.910)	< 0.001	3.829 (1.662,9.071)	0.002
Sputum smears positive	2.091 (1.281,3.428)	0.003	2.222 (1.086,4.623)	0.03
2 months smears positive	3.475 (1.210,10.703)	0.022	3.416 (1.133,10.910)	0.03
Xpert positive	1.935 (1.178,3.255)	0.01	1.958 (1.109,3.523)	0.022
TB-DNA positive	2.358 (1.440,3.880)	< 0.001	2.582 (1.422,4.756)	0.002
Negative conversion rate	0.212 (0.07,0.642)	0.006	0.249 (0.082,0.758)	0.014
Cough	2.262 (1.311,4.031)	0.004	0.742 (0.241,2.106)	0.586
Expectoration	2.261 (1.382,3.749)	0.001	2.023 (0.755,5.891)	0.174
Concomitant bronchial TB	10.616 (4.156,32.706)	< 0.001	23.126 (7.278,92.451)	< 0.001
Concomitant extrapulmonary TB	2.793 (1.228,6.349)	0.014	2.972 (1.302,6.788)	0.01
Lung fields ≥3	1.752 (1.462,2.127)	< 0.001	1.718 (1.406,2.128)	< 0.001
Cavity	1.949 (1.193,3.194)	0.008	2.564 (1.469,4.527)	< 0.001
3 months treatment outcome				
Significant favorable	2.521 (1.413,5.513)	0.002	1.998 (1.065,3.731)	0.029
Favorable	4.042 (1.602,10.574)	0.003	3.306 (1.233,9.047)	0.017
Unfavorable	2.131 (0.272,13.346)	0.417	1.993 (0.249,12.868)	0.466
6 months treatment outcome				
Significant favorable	0.464 (0.178,1.065)	0.088	0.447 (0.149,1.164)	0.12
Favorable	3.854 (0.978,1.023)	0.063	3.578 (0.897,19.265)	0.08
Unfavorable	/		/	

*P* < 0.05 is statistically significant. TB, tuberculosis; CI, confidence interval

### Plasma sCD25 levels decreased in group TB2 after anti-TB treatment

**Fig. 3:**
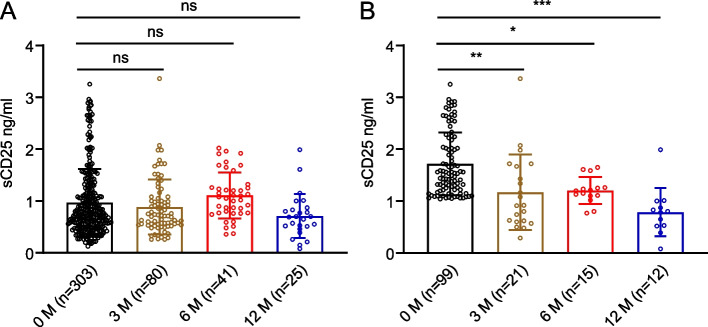
Changes in plasma sCD25 levels following anti-TB treatment. **A** Plasma levels of sCD25 in TB patients after anti-TB treatment. **B** Plasma levels of sCD25 in Group TB2 patients after anti-TB treatment. 0 M, TB patients before the anti-TB treatment; 3, 6, and 12 M, TB patients after 3, 6, and 12 months of the anti-TB treatment. Data are mean ± SD, * *P* < 0.05, ** *P* < 0.01, *** *P* < 0.001

The plasma sCD25 levels of the patients after anti-TB treatment are shown in Fig. 3. Compared with patients before anti-TB treatment, the levels of sCD25 were slightly lower at 3 and 12 months of anti-TB treatment, although there was no significant difference (Fig. 3A). Furthermore, in Group TB2, there was a progressive reduction of the plasma sCD25 levels after anti-TB treatment (Fig. 3B), which were significantly lower than those before treatment. The levels were 1.171 ± 0.730, 1.205 ± 0.258, and 0.788 ± 0.464 ng/ml at 3, 6, and 12 months, respectively, after anti-TB treatment. In Group TB1, however, not a progressive reduction was observed (Additional file 1: Fig. S2). These data indicate that the plasma levels of sCD25 was a sensitive index of response to anti-TB treatment.

### Analysis of typical cases

**Fig. 4:**
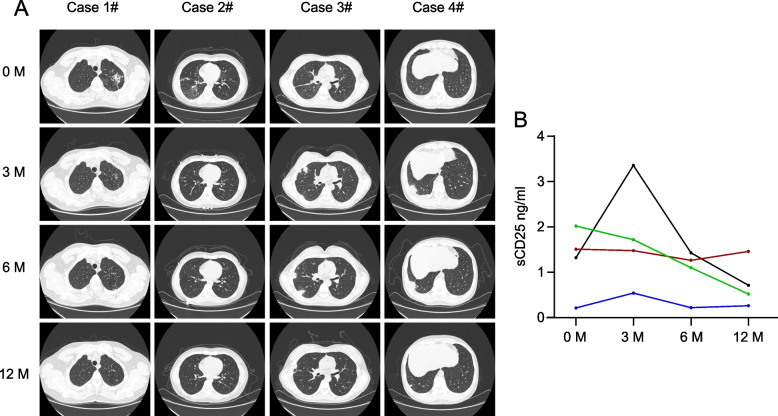
CT imaging and levels of plasma sCD25 in TB patients. **A** CT imaging of four typical patients with before and after anti-TB treatment. **B** Levels of sCD25 before and after anti-TB treatment in TB patients. 0 M, TB patients before the anti-TB treatment; 3, 6, and 12 M, TB patients after 3, 6, and 12 months of the anti-TB treatment

We selected typical TB cases to analyze the correlation between chest CT imaging and the levels of plasma sCD25 (Fig. 4 and Additional file 1: Fig. S3). Case 1 and Case 5: The levels of plasma sCD25 were high before treatment, and gradually decreased after anti-TB treatment. At the same time, the initial chest CT imaging of the patient showed a lung lesion in the left upper lobe. Subsequently, this lesion was absorbed with the decrease of extent and density after 3 months of anti-TB treatment. On the latest chest CT (12 months later), the lesion was completely absorbed. Case 2 and Case 6: The patient showed low levels of plasma sCD25 before and after anti-TB treatment, and his lung lesions distributed in relatively fewer fields. However, Case 3 and Case 7: The plasma sCD25 was at a high level before and after anti-TB treatment. Along with that, the patient did not achieve the desired therapeutic effect, and presented lesions of multiple lung fields. Case 4 and Case 8: With anti-TB treatment, the plasma sCD25 levels of the patient increased within 3 months and decreased during the following period. We found that the lung lesions of the patient progressed significantly in the third month of treatment. Case 8 patient had cavity formation after anti-TB treatment 3 months. With the decrease of sCD25 levels in the patient, the corresponding lung lesions were also gradually absorbed. These results suggest that the levels of plasma sCD25 are associated with the absorption or progression of lung lesions.

## Discussion

TB remains to be one of the deadliest infectious diseases in the world, from which every 20 seconds a person dies [22]. The occurrence, progress, and prognosis of TB are related to the host immune status. Measuring the T-cell activation would be helpful to assess the TB patients’ immune status. sCD25 is considered to be a substitute marker and indicator for T cell activation. Studies carried out in Taiwan showed that sCD25 levels appear to be clinically useful as a biochemical marker to differentiate TB and carcinomatous pleural effusions [23]. In a cohort of TB patients in Hong Kong, the serum sCD25 values were markedly elevated in patients with pulmonary TB (parenchymal lesion and pleural effusion) compared with old inactive TB patients, and normal control subjects [24]. In our study, we found that sCD25 levels were significantly elevated in the plasma of untreated TB patients, which were consistent with previous reports [25]. Elevated plasma sCD25 indicates that the immune system was activated in those patients. The levels of plasma sCD25 fell progressively at 3, 6, and 12 months after anti-TB treatment, but the trend did not attain statistical significance probably because of the small sample size. Plasma measurements of sCD25 may be used to evaluate the disease phase and the response to treatment.

A ROC curve using a cut-off value of 1.037 demonstrated the potential of plasma sCD25 as a sensitive biomarker for TB risk, with 32.67% sensitivity, 96.49% specificity, and an area under the ROC curve (AUC) of 0.605. The cut-off value determines the test sensitivity and specificity. However, for a given test, we cannot increase the sensitivity and specificity concomitantly. Specificity will be enhanced at the expense of sensitivity and vice versa. In the clinic, we may pay more attention to high sensitivity to avoid misdiagnosis and unnecessary intervention and treatment.

The core goal of ROC curve analysis is to determine a reasonable cutoff value for sCD25 to aid the possibility of diagnosing TB. It is worth noting that although the resulting AUC of 0.605 does not fully represent excellent diagnostic performance, it provides an important clue for the preliminary investigation of the relationship between sCD25 levels and TB. From a statistical point of view, this AUC value is not a strong differentiator, but from a clinical point of view, this preliminary finding may provide clinicians with an additional reference information when faced with fuzzy diagnosis situations.

In this study, those patients whose plasma sCD25 levels were above the cut-off threshold had a significantly reduction of the plasma sCD25 levels after anti-TB treatment. These results are consistent with the finding conducted in Hong Kong [26]. This decline may be due to the recovery of the immune status of TB patients after the therapy. Therefore, the measurement of plasma sCD25 may be helpful in monitoring the occurrence and progress of TB and the host immune response during therapy.

The typical symptoms of pulmonary TB include fever, night sweats, abnormal fatigue, cough, and hemoptysis. In the current study, we analyzed the association of plasma sCD25 levels with clinical, laboratory, and CT imaging characteristics of TB patients. Symptoms of TB patients in Group TB2 appeared to be more common and severe than those described in Group TB1. Patients in Group TB2 showed a high incidence in cough, expectoration, and positive MTB test results. Previous studies showed that there was a positive correlation between serum sCD25 values and the extent of disease on chest radiograph [24]. In our study, the presence of cavities and lesions of multiple lung fields were more common among Group TB2 patients. All these results may contribute to unfavorable treatment outcomes in Group TB2.

In 2010, the WHO guidelines recommended to treat drug-susceptible TB (DS-TB) patients with a 6-month regimen composed of four first line TB medicines. Although 85% of patients could successfully complete the course of treatment, the rest found that the 6-month regimen difficult to complete due to its duration [27]. In fact, long treatment regimens present serious challenges both to the patients and to the programmatic management of TB globally. Recently, WHO recommended patients with DS-TB to receive a 4-month short regimen [27]. This indicates that it is important to predict the efficacy of TB treatment as soon as possible, and identify which patients are suitable for the short treatment regimens. In our study, there was a significant difference of clinical outcomes between Group TB1 and Group TB2 after the therapy. Interestingly, there was a high proportion of favorable outcomes in Group TB1 and unfavorable outcomes in Group TB2. The condition of most TB patients could be controlled after the treatment intensification. However, there were also some patients who could not achieve the expected therapeutic effect (for example, sputum bacteria were positive and/or lung lesions were still in progress) after intensification period. These results suggest that the levels of plasma sCD25 are negatively correlated with the outcomes of anti-TB treatment, and patients with low levels of sCD25 in plasma may be more suitable for a 4-month short regimen.

Studies had confirmed that urinary sCD25 seems to be a good biomarker for the follow-up of lupus nephritis patients, with the potential to predict relapse and response to treatment [28]. In the typical cases we analyzed, patients with high plasma sCD25 levels had more severe lung lesions. To some extent, the plasma sCD25 levels may be a biomarker of clinical outcome in TB patients, which could reflect the progression and prognosis of TB. Collectively, the measurement of plasma sCD25 may be used to evaluate the condition of TB patients and predict the efficacy of anti-TB treatment, and identify which patients are suitable for the 4-month short regimen. It can not only shorten the treatment course of the patients, promote patient compliance, improve the treatment efficiency, but also reduce the economic burden of patients.

However, there are still several limitations to this study. Firstly, the number of TB patients in each clinical group was relatively small, which might have limited the detection of significant associations, especially during follow-up, when the number of participants further decreased. Secondly, we could not identify a bias of interobserver variability in the clinical signs and the diagnostic approach of each case. Finally, it should be pointed out that our study focused on the correlation between plasma sCD25 levels and conditions of TB patients but was not mechanism oriented. In spite of these limitations, this study provides new insight to the study on the role of sCD25 in TB patients, which is of promising clinical application significance when used in evaluation of disease severity, progression, and prognosis of TB.

## Conclusions

In this study, we found that sCD25 levels were significantly elevated in the plasma of untreated TB patients. Furthermore, those patients whose plasma sCD25 levels were above the cut-off threshold had a significantly reduction in the plasma sCD25 levels after anti-TB treatment. In addition, these patients showed a high percentage in cough, expectoration, positive MTB test results, and lesions of lung fields. Besides, they exhibited an increased proportion of unfavorable outcomes after anti-TB treatment. The clinical, laboratory and radiological manifestations of TB patients with high plasma sCD25 levels indicate that the TB disease is more severe.

### Supplementary Information

**Additional file 1.**

## Data Availability

The datasets used and/or analyzed during the current study are available from the corresponding author on reasonable request.

## Abbreviations

CT: Computed Tomography
DS-TB: Drug-susceptible tuberculosis
ELISA: Enzyme linked immunosorbent assay
HC: Healthy controls
IQR: Interquartile range
MTB: *Mycobacterium tuberculosis*
ROC: Receiver operator characteristic
SD: Standard deviation
TB: Tuberculosis
