# Supplementary material for: The clinical significance of plasma sCD25 as valuable biomarker for progression and prognosis of tuberculosis
Source: BMC Infect Dis. 2024 Jan 22;24:115. doi: 10.1186/s12879-023-08798-5 (PMC10804724; doi:10.1186/s12879-023-08798-5)
Supplement: Supplementary file 1 — Additional file 1. [file 12879_2023_8798_MOESM1_ESM.doc]

**Figure S1. ROC curve of plasma sCD25 levels.** Receiver operating characteristics (ROC) curve was used to evaluate the sCD25 for the prediction of TB risk. Dashed lines supposedly indicate the confidence intervals.

**Figure S2. Plasma levels of sCD25 in Group TB1 patients.** Plasma levels of sCD25 in Group TB1 patients after anti-TB treatment. 0 M, TB patients before the anti-TB treatment; 3, 6, and 12 M, TB patients after 3, 6, and 12 months of the anti-TB treatment. Data are mean ± SD, *** P < 0.001. ns, no significance.

**Figure S3. CT imaging of four typical patients with before and after anti-TB treatment.** 0 M, TB patients before the anti-TB treatment; 3, 6, and 12 M, TB patients after 3, 6, and 12 months of the anti-TB treatment.

**Table S1.** Potential of sCD25 as a Biomarker of TB.

| Factor | AUC | Cut-off | Sensitivity | Specificity | Youden index | *P* |
| --- | --- | --- | --- | --- | --- | --- |
| CD25 | 0.605 | 1.037 | 32.67 | 96.49 | 0.292 | 0.001 |
